# Supplementary material for: Mice in a labyrinth show rapid learning, sudden insight, and efficient exploration
Source: eLife. 2021 Jul 1;10:e66175. doi: 10.7554/eLife.66175 (PMC8294850; doi:10.7554/eLife.66175)
Supplement: Figure 5—source data 1. — Statistics of sudden changes in behavior. Summary of the steps in the rate of long paths to water detected in 5 of the 10 rewarded animals. Mean and standard deviation of the step time are derived from maximum likelihood fits of a step model to the data. [file elife-66175-fig5-data1.pdf]

Steps in the rate of long paths

Animal	Time of step (s)	Ratio of rates after/before
**B1**	2580 ± 110	36.4
**B2**	2350 ± 220	30.3
**C1**	2070 ± 310	5.49
**C3**	1280 ± 80	1640
**C7**	1680 ± 280	16.9
