# Supplementary material for: Mice in a labyrinth show rapid learning, sudden insight, and efficient exploration
Source: eLife. 2021 Jul 1;10:e66175. doi: 10.7554/eLife.66175 (PMC8294850; doi:10.7554/eLife.66175)
Supplement: Figure 7—source data 1. — (A) The fraction of time mice spent in each of the three modes while in the maze. Mean ± SD for 10 rewarded and nine unrewarded animals. (B) Probability of transitioning from the mode on the left to the mode at the top. Transitions from ‘leave’ represent what the animal does at the start of the next bout into the maze. [file elife-66175-fig7-data1.pdf]

A

Fraction of time in modes

Mode	rewarded	unrewarded
**leave**	0.053 ± 0.014	0.054 ± 0.013
**drink**	0.103 ± 0.026	
**explore**	0.844 ± 0.032	0.946 ± 0.013

B

Transition probability between modes

from/to:	leave	drink	explore
**leave**		0.51 ± 0.14	0.49 ± 0.14
**drink**	0.10 ± 0.05		0.90 ± 0.05
**explore**	0.40 ± 0.11	0.60 ± 0.11	
