# Supplementary material for: Mice in a labyrinth show rapid learning, sudden insight, and efficient exploration
Source: eLife. 2021 Jul 1;10:e66175. doi: 10.7554/eLife.66175 (PMC8294850; doi:10.7554/eLife.66175)
Supplement: Figure 9—source data 1. — Statistics of the four turning biases. Mean and standard deviation of the 4 biases of Figure 9A–B across animals in the rewarded and unrewarded groups. [file elife-66175-fig9-data1.pdf]

Bias statistics

Bias	rewarded	unrewarded
P_{SF}	0.77 ± 0.03	0.78 ± 0.02
P_{SA}	0.72 ± 0.02	0.71 ± 0.02
P_{BF}	0.82 ± 0.03	0.81 ± 0.03
P_{BS}	0.64 ± 0.02	0.63 ± 0.02
